# Parameterization of magnetic vector potentials and fields for efficient multislice calculations of elastic electron scattering

**DOI:** 10.1107/S2053273321008792

**Published:** 2021-10-29

**Authors:** Keenan Lyon, Jan Rusz

**Affiliations:** a Department of Physics and Astronomy, Uppsala University, Box 516, S-751 20 Uppsala, Sweden

**Keywords:** magnetism, multislice, parameterization, simulation, DFT

## Abstract

The multislice method, which simulates the propagation of the incident electron wavefunction through a crystal, is a well established method for analysing the multiple scattering effects that an electron beam may undergo. The inclusion of magnetic effects into this method proves crucial towards simulating enhanced magnetic interaction of vortex beams with magnetic materials, calculating magnetic Bragg spots or searching for magnon signatures, to name a few examples. Inclusion of magnetism poses novel challenges to the efficiency of the multislice method for larger systems, especially regarding the consistent computation of magnetic vector potentials **A** and magnetic fields **B** over large supercells. This work presents a tabulation of parameterized magnetic (PM) values for the first three rows of transition metal elements computed from atomic density functional theory (DFT) calculations, allowing for the efficient computation of approximate **A** and **B** across large crystals using only structural and magnetic moment size and direction information. Ferromagnetic b.c.c. (body-centred cubic) Fe and tetragonal FePt are chosen to showcase the performance of PM values versus directly obtaining **A** and **B** from the unit-cell spin density by DFT. The magnetic fields of b.c.c. Fe are well described by the PM approach while for FePt the PM approach is less accurate due to deformations in the spin density. Calculations of the magnetic signal, namely the change due to **A** and **B** of the intensity of diffraction patterns, show that the PM approach for both b.c.c. Fe and FePt is able to describe the effects of magnetism in these systems to a good degree of accuracy.

## Abstract 2

A tabulation is presented of parameterized magnetic fields computed from atomic density functional theory calculations that allows for the efficient computation of approximate magnetic vector fields in materials using only structural and magnetic moment size and direction information. Multislice calculations of the change in the intensity of diffraction patterns due to magnetism for body-centred cubic Fe and FePt show that this approach is able to describe the effects of magnetism in these kinds of systems to a good degree of accuracy.

## 1. Introduction   

The engineering, design and exploration of novel magnetic materials necessitate characterization methods capable of rendering the behaviour of these materials down to the atomic scale. Recent progress in the development of electron beam monochromators has made it possible for the ever-versatile transmission electron microscope to probe low-energy excitations at this scale. Detection of magnetism in samples remains challenging, given that the interaction of magnetic moments with the electron beam is weaker than the Coulomb interaction by three to four orders of magnitude (Chapman *et al.*, 1978 ▸; Rother & Scheerschmidt, 2009 ▸; Loudon, 2012 ▸). Within the transmission electron microscope setup, electron holography (Tonomura, 1995 ▸), Lorentz microscopy (McVitie *et al.*, 2015 ▸), differential phase contrast microscopy (Edström *et al.*, 2019 ▸) and electron magnetic circular dichroism (Schattschneider *et al.*, 2006 ▸) have all been put forward as approaches to study magnetism in materials. As these approaches gain momentum in the literature (McVitie *et al.*, 2015 ▸; Schattschneider *et al.*, 2006 ▸), there is a clear need for a consistent and efficient description of magnetic vector potentials and fields in the materials under consideration. Efficiency becomes key in simulations of crystalline systems used in electron microscopy, where crystals on a size scale beyond the reach of standard density functional theory (DFT) or other commensurate methods limit the computational capability for describing magnetism from *ab initio* methods directly.

Such efficiency becomes a moot point if the effects of magnetism cannot be readily measured in an experimental setup. To this end, magnetic effects in the elastic scattering regime can reach relative strengths of up to a few per cent (Edström *et al.*, 2016*a* ▸,*b* ▸, 2019 ▸) through the use of phase-shaped electron beams, such as aberrated or vortex beams (Bliokh *et al.*, 2017 ▸; Schattschneider *et al.*, 2014 ▸), although gradual deterioration of the angular momentum of the electron beam tends to occur as it traverses the crystal (Löffler *et al.*, 2019 ▸; Lubk *et al.*, 2013 ▸; Rusz *et al.*, 2014 ▸). Furthermore, modern direct and hybrid-pixel detectors currently offer drastically improved detection dynamic range and low background noise, with detection capability close to 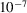 of the full beam intensity within tens of pixels of the recorded signal’s maximum (Plotkin-Swing *et al.*, 2020 ▸). With recent improvements in monochromator and spectrometer design, resulting in increased energy resolutions, especially at lower acceleration voltages (Krivanek *et al.*, 2019 ▸), signals of weak intensity such as those related to magnetic effects are within the realm of experimental feasibility, as shown by recent work towards the detection of such effects in antiferromagnetic materials (Huang *et al.*, 2021 ▸; Loudon, 2012 ▸).

When it comes to a parameterization of a potential in the context of the multislice method, electron atomic scattering factors (Doyle & Turner, 1968 ▸; Weickenmeier & Kohl, 1991 ▸; Peng, 1999 ▸, 2005 ▸; Kirkland, 2010 ▸; Lobato & Van Dyck, 2014 ▸), first introduced to describe and evaluate the scattered beam amplitudes of electrons by crystals, come to mind. The use of the electron atomic scattering factors in this context relies on two main assumptions, namely that incoming electrons travelling at high enough energies will see the atom as a scattering centre, and that the total Coulomb potential can be computed as a superposition of atomic potentials, neglecting the charge redistribution that occurs in a crystal. While not identical, if similar criteria are assumed to hold true for magnetic fields and vector potentials with certain limitations, it stands to reason that knowledge of these quantities for an atomic setup can be used in superposition to build up a suitable approximation for the magnetic profile of any material. As with electron atomic scattering factors, it is important to note that bonding in many materials has a considerable effect on the spin densities of the valence electrons, and the advantage presented through use of an independent atom approximation to magnetism is only as useful as the quality of such an approximation to the system under consideration.

For such a parameterization to be generally useful across a large spectrum of systems of interest in electron microscopy, certain criteria must be met. First, the difference in the atomic magnetic moment for the same atom in different crystal configurations must be easy to account for. Second, the potentials and fields should be smoothly varying, independent of the choice of grid on which the quantities are represented. Third, for computational efficiency, the determination of magnetic quantities at a given grid point should depend solely on local structural and magnetic moment size and direction information. This paper presents the quasi-dipole approach, satisfying all of the above criteria and thereby streamlining the implementation of magnetic potentials and fields into the growing set of methods in microscopy that take account of magnetic effects in materials (Negi *et al.*, 2018 ▸; Edström *et al.*, 2019 ▸; Lyon *et al.*, 2021 ▸; Krizek *et al.*, 2020 ▸; Kovács *et al.*, 2017 ▸; Schneider *et al.*, 2018 ▸; Midgley & Dunin-Borkowski, 2009 ▸; Grillo *et al.*, 2017 ▸; Matsumoto *et al.*, 2016 ▸; Chen *et al.*, 2018 ▸; Nguyen *et al.*, 2020 ▸; Verbeeck *et al.*, 2010 ▸). In this work, specific use is made of the Pauli multislice method (Edström *et al.*, 2016*a* ▸,*b* ▸), which employs the paraxial Pauli equation in a multislice formalism to account for the role of **A** and **B** fields in electron beam scattering.

The methodology behind the parameterized magnetism method is outlined in Section 2, including the calculation of the periodic components of **A** and **B** from the spin density in DFT, the quasi-dipole approximation that forms the basis for parameterization of atomic magnetic components, and the computational details of the DFT calculations and the least-squares fit. Tabulated results for 25 transition metals are given in Section 3.1, while the quality of the parameterized magnetism approach is benchmarked against DFT results and against different grids and geometries in Section 3.2. Section 3.3 concludes the paper by showing how the parameterized magnetism approach utilized in the Pauli multislice method compares with DFT results. This comparison is done by calculation of the magnetic signal, namely the redistribution of intensity in diffraction patterns due to the periodic components of **A** and **B**, as measured via the squared amplitude of the exit wavefunction in a multislice setup.

## 2. Methodology   

In the following sections we will summarize the methods used in this work. This begins in Section 2.1 with a summary of how the magnetic vector potential 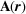 and magnetic field 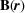 can be calculated using DFT in a consistent manner, as shown by Rother & Scheerschmidt (2009 ▸), Edström *et al.* (2016*a* ▸). The framework for the parameterization of fields surrounding atoms by fitting to DFT calculations is then developed in Section 2.2. Computational details are summarized in Section 2.3.

### 2.1. Calculation of **A** and **B** fields   

The magnetization density for a crystalline system is given by 

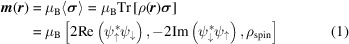

where 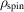 = 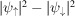 is the spin density projected onto the spin quantization axis (Edström *et al.*, 2016*b* ▸). For the case of atomic systems, collinear magnetism occurs by default, resulting in the simplified 

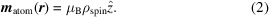

In order to obtain the magnetic vector potential **A** and the corresponding flux density **B**, following Edström *et al.* (2016*b* ▸), we first make the assumption that for the materials under consideration in this paper we can safely neglect the orbital current density. While this assumption is not valid for all elements, especially for *f*-electron systems such as rare earths and actinides, quenching of orbital angular momentum due to interaction with the crystalline electric field applies for the substantial majority of transition metals with unfilled *d*-electron shells (Mohn, 2006 ▸). The total current density can therefore be expressed via (Strange, 1998 ▸) 

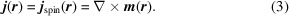

Working with Maxwell’s equations in the Coulomb gauge ( 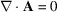 ) yields 

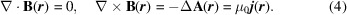

Applying the Fourier transform over the Brillouin zone (BZ) to the magnetic field in real space, 

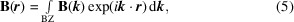

gives 

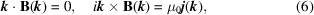

where 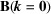 corresponds to the volume average of 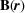 . As we seek to calculate **A** and **B** fields on a grid of a unit cell, where the magnetization density is defined, it is necessary to separate the periodic components ( 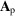 , 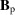 ) from the non-periodic ones ( 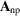 , 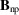 ). Considering that, by definition, 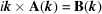 , a periodic vector potential 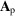 can only be achieved by setting the 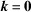 component of the periodic magnetic field, 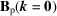 , equal to zero, corresponding to a zero average magnetic field in the unit cell (Edström *et al.*, 2016*b* ▸). The non-periodic component 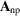 , corresponding to the average magnetic field within the unit cell, is defined in the Coulomb gauge via 

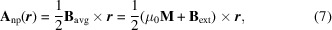

where **M** is the macroscopic magnetization of the material and 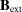 is an external magnetic field. Since 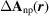 is here equal to zero, equation (4) can be written as 

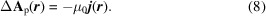

Applying the Fourier transform over the BZ to the periodic part of **A** allows, in conjunction with the Fourier transform of equation (3), for equation (8) to be rewritten as 

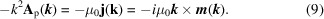

The magnetic vector potential and magnetic field can thereby be expressed in reciprocal space as 

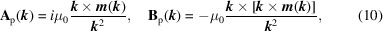

where 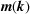 is the 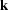 component of the Fourier-transformed atomic magnetization [see equation (1)]. The average of 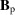 , as mentioned before, is zero due to the requirement of the periodicity of the corresponding 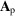 , while the average of 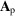 in equation (10) can be chosen to be zero by gauge freedom (Jackson, 1999 ▸). For a DFT calculation where 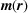 is defined on a grid, applying forward and backwards Fourier transforms will directly yield the periodic components of 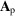 and 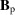 in real space, with the zero average condition enforced by setting 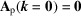 and 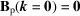 .

### 2.2. Quasi-dipole approximation   

In order to describe the magnetic vector potential and fields for each atomic system, we seek a function which can be parameterized in a way similar to electron form factors while still retaining the main properties of the dipole-like fields that surround atoms, a property that naturally comes about due to Hund’s rule for the maximization of spin (Strange, 1998 ▸). For this approach we opt for a quasi-dipole formulation, such that the magnetic vector potential **A** and magnetic field 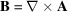 are defined via 

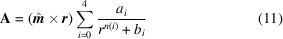




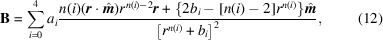

where 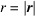 , 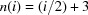 and 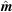 is the unit vector with direction of the magnetic moment of the atom. The 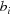 coefficients in this equation serve to smooth out the short-range behaviour of the classical dipole, while the 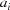 modulate the strength of the field over all space. The choice of five pairs of parameters, corresponding to ten total coefficients, was necessary to achieve the desired root mean square (RMS) error for the **B** field below 0.1 T across all elements under consideration. Having the summation stop at order 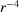 for the magnetic vector potential ( 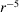 for the magnetic field) serves to prevent high-frequency oscillations that may be expected to occur at higher orders. Lastly, the choice of five pairs of parameters helps to ensure the drop-off of magnetic field strength beyond 3 Å from the atomic centre. It is important to note that the magnetic moment of an atom changes its magnitude depending on the surrounding environment (Billas *et al.*, 1994 ▸), so considering materials with variable magnetic moments necessitates a rescaling of the magnetic moment vector.

Utilizing the above equations serves three main purposes. First, introducing the parameters 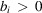 eliminates the possible divergence associated with using a traditional dipole approximation. Second, by allowing for a sum over higher powers of the radial distance in the denominator, this approximation is better able to capture the short distance fluctuations within 1 Å of the atomic centres that differentiate the magnetic properties of each element. Third, the shape of **A** and **B** will remain the same no matter which way the moment points, and the parameterizations for each element can readily be used in the computation of larger structures, as only the element type and the direction and size of the magnetic moment are necessary to yield the periodic **A** and **B** components over all space. In addition, the symmetry of the fit function allows for the computation of radial prefactors that drastically speed up computation speed, namely 

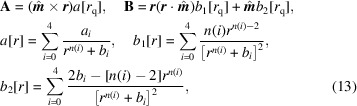

where 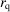 is a discretized approximation to *r*, depending on the grid spacing of the radial prefactors.

One major aspect where the atomic and bulk magnetic quantities diverge is in the quashing of the spin magnetic moment in the transition from atomic systems to clusters to the bulk (Billas *et al.*, 1994 ▸), a natural consequence of the delocalization of atomic orbitals in response to bonding. This decreased local magnetic moment means that each individual atom will contribute less to the total **A** and **B** than in the atomic case. While a proper treatment of the shape of magnetic fields in response to orbital deformation needs to be considered on a material-specific basis, for the purposes of general approximation we present in this work that a simple scaling of the atomic parameterized fields to the experimental magnetic moment values is sufficient for the purposes of multislice calculations.

In order for this parameterization to be generally useful towards the approximation of **A** and **B** for crystals of arbitrary size, the condition that the **A** and **B** within a certain area of each atom sum to zero is crucial, as this allows for the periodic component of **A** and **B** to be directly constructed from the individual zero-average atomic magnetic components. As seen in equation (7), the total magnetic field and vector potential for a system can then be obtained using the total magnetization. However, it is important to note that calculation of the parameterized values 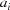 and 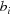 in equations (11) and (12) for atomic systems are optimized on a specific fine grid, so care must be taken that the final total sum of the periodic components of **A** and **B** over the entire supercell is as close to zero as possible.

### 2.3. Computational details   

All DFT calculations employ the projector augmented wave method code *GPAW* (Mortensen *et al.*, 2005 ▸; Enkovaara *et al.*, 2010 ▸) within the atomic simulation environment *ase* (Bahn & Jacobsen, 2002 ▸; Larsen *et al.*, 2017 ▸). An electronic temperature of 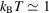 meV was chosen. All calculations are done in the spin polarized state. The Kohn–Sham wavefunctions are represented by plane waves (PWs) with a converged energy cutoff of 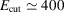 eV. For the 25 atomic calculations, Gamma point calculations were performed with a unit cell of dimensions 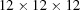 Å. The Perdew–Burke–Ernzerhof (PBE) parameterization (Perdew *et al.*, 1996 ▸) of the generalized gradient approximation to the exchange-correlation (XC) functional was chosen for every element except for scandium, iron, nickel, rhodium and osmium, for which the closely related PBEsol (Perdew *et al.*, 2008 ▸) XC functional was chosen due to convergence issues. The magnetic moments for scandium and palladium were forced into the 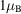 and 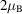 states, respectively, as otherwise these atomic systems proved difficult to converge. For b.c.c. (body-centred cubic) iron, defined by a 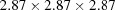 Å unit cell with iron atoms located at 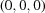 and 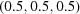 in scaled coordinates, and for FePt (Gilbert *et al.*, 2013 ▸), defined by a 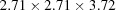 Å unit cell with iron at 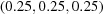 and platinum at 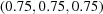 in scaled coordinates, the PBE XC functional was again chosen while using a 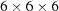 *k*-point Monkhorst–Pack mesh (Monkhorst & Pack, 1976 ▸) for both calculations, noting that the relatively small *k*-point grid is sufficient for describing the approximate electron density in these structures. The local magnetic moment calculated in *GPAW* for Fe in the b.c.c. iron unit cell is 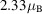 , while in the FePt unit cell the local moment for Fe is 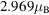 and for Pt is 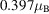 .

To simulate the orientation of magnetic moments for a supercell of b.c.c. Fe of size 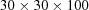 unit cells in response to thermal fluctuations, angles θ for the magnetic moment divergence from the *z* axis were sampled from a multivariate normal distribution of mean zero (*i.e.* aligned with the *z* axis) and standard deviation 30°, simulating a supercell with 90% of the *z*-direction magnetization of a collinear supercell, while the azimuthal angles ϕ were sampled uniformly from 0 to 360°. An exponential distance decay factor (Wackernagel, 2003 ▸; Rusz *et al.*, 2006 ▸) of 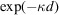 , where 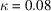 Å^−1^ and *d* is the distance between spins in ångström, was introduced into the covariance matrix for both distributions to imitate in-plane spin–spin spatial correlation, while spatial correlation along the *z* axis was imitated by doing a layer-by-layer iterative mixing of θ and ϕ, so every layer consists of a weighted average of 2/3 the angles from the layer above and 1/3 the angles drawn from the multivariate distributions.

**A** and **B** generated from the atomic DFT calculations yield grids of 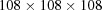 points over the 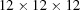 Å cells. The parameterized values in equations (11) and (12) are obtained with the *LMFIT* (Newville *et al.*, 2021 ▸) package in Python, with optimization carried over the approximately 23 000 points within a 2 Å radius of the atom centre and an additional 47 000 points randomly chosen from elsewhere in the unit cell, with the only restriction on the least-squares fit being that 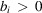 for all materials. An RMS error calculation is evaluated over the 70 000 total points involved in the calculation of the fit. RMS errors of at most 0.1 T for the fits to **B** and 0.025 T Å for the fits to **A** were obtained.

For all calculations making use of the parameterized **A** and **B** fields, a cutoff radius of 3 Å around each atom was used, with the contribution of the atom beyond this radius to the **A** and **B** fields set to zero.

## 3. Results and discussion   

In the following sections we present the results of this work. The tabulation of the parameterized magnetization values is shown in Section 3.1. Section 3.2 shows a simulation for the appearance of the magnetic vector potential on a large supercell, examines the performance of the parameterized magnetism approach compared with DFT in describing magnetic fields in unit cells, and considers the flexibility of the approach with different grid sizes and geometries. Finally, Section 3.3 compares the magnetic signal from both a b.c.c. iron and a tetragonal FePt supercell, using magnetic fields and potentials determined by the parameterized approach versus DFT as input to a Pauli multislice approach.

### 3.1. Tabulated magnetic coefficients for the transition metals   

Table 1 ▸ shows the parameterized magnetic factors for transition metal elements from scandium ( 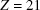 ) to gold ( 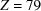 ), following equations (11) and (12), neglecting elements with filled *d* orbitals (and therefore zero spin magnetic moment) or for which *GPAW* has no atomic projector augmented wave (PAW) setups (Tc). The parameters 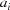 are scaled so that the resulting **A** and **B** values correspond to an atom with spin magnetic moment of one Bohr magneton (μ_B_). Therefore, for example, calculations for a b.c.c. iron supercell would involve a rescaling of the listed 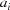 parameters in Table 1 ▸ by a factor of 2.33 for every constituent iron atom.

**Figure 1:**
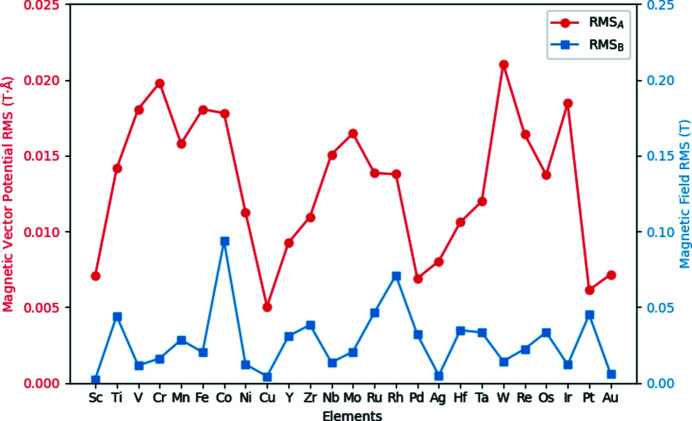
RMS error for the least-squares fit of parameterized values in equations (11) and (12) versus atomic DFT-calculated **A** and **B** values.

Three main features stand out from Table 1 ▸. First is the fact that the values of 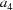 , corresponding to a quasi-dipole that propagates asymptotically in space as 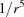 rather than the 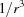 of the classical dipole (Jackson, 1999 ▸), have a median an order of magnitude lower than values 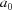 to 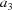 , suggesting that the fluctuations in the magnetic fields located very close to the atomic centres are not crucial to the overall performance of the model. Second is that all values 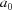 are positive across all elements, which matches with the expectation that since these coefficients correspond to the quasi-dipole term closest to the classical dipole, the contribution to the overall magnetization is positive as well. Third, the median values for 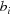 are between 0.25 and 1.25 in units of Å^*n*(*i*)^, suggesting again that on the whole no one term in the quasi-dipole approximation is accounting for short- or long-term behaviour of the **A** and **B** values over the entire unit cell. The RMS error for the calculations over the 25 transition metal elements is presented in Fig. 1 ▸. No general relationship exists between parameterizations that yield good fits for **A** while also doing so for **B**, but most importantly the maximum error of this approach is revealed to be consistent across a range of atomic elements.

### 3.2. Evaluation of the parameterized magnetism approach   

**Figure 2:**
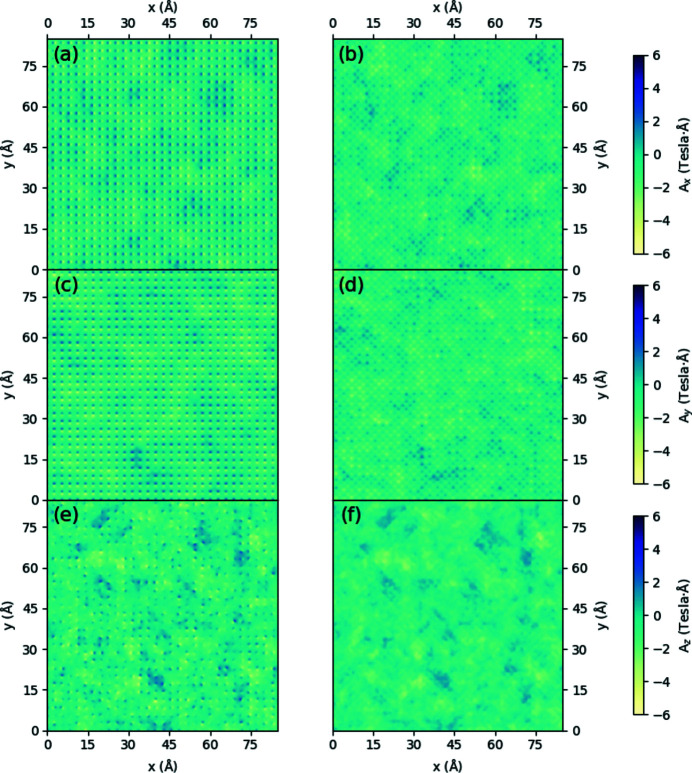
Heatmaps in the *xy* plane for (*a*), (*b*) 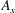 , (*c*), (*d*) 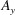 and (*e*), (*f*) 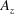 for a supercell of b.c.c. Fe of size 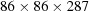 Å, with magnetic vector potential generated from the parameterization of magnetic fields around the iron atom. Directions of the magnetic moments are given by spatially correlated multivariate normal and uniform distributions. (*a*), (*c*), (*e*) are for the plane located at *z* = 0 Å, where 900 atoms lie at the surface, while (*b*), (*d*), (*f*) are for the plane located at *z* = 0.75 Å deeper in the material.

To showcase the capabilities of the parameterized magnetism (PM) approach, Fig. 2 ▸ presents heatmap plots in the *xy* plane for three components of **A** for a supercell of b.c.c. Fe of size 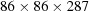 Å, *i.e.* 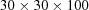 unit cells. The orientation of the magnetic moments, meant to provide a basic simulation of thermal fluctuations, is given by selecting two angles for each moment from spatially correlated multivariate distributions, as explained in Section 2.3. The magnetic field and vector potential were evaluated on a grid of 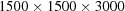 points, utilizing the parameterization of the **A** and **B** fields for the iron atom and rescaling to the 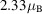 magnetic moment of b.c.c. Fe. In Fig. 2 ▸, the subfigures in the left column are for the plane located at *z* = 0 Å, where 900 atoms lie at the surface. A sort of grid-like pattern emerges for 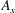 and 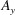 , as nearly all moments are oriented towards the *z* axis and the fluctuations in the spin density are strongest nearest to the atoms. Fewer of these punctures are visible for 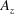 as the local moments there must point relatively off the *z* axis. A broad continuity of the colour spectrum is also visible, reflecting the slow fluctuations expected from the chosen spatial autocorrelation factor. The lack of sharp peaks or troughs in the vector potential is a reflection of the introduction of 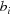 terms in equations (11) and (12), which would not be the case for certain choices of grid in a classical dipole approach.

The right column subfigures of Fig. 2 ▸ are for the plane located at *z* = 0.75 Å deeper in the material. It is evident that the general fluctuations in the three components of **A** match with those of the left column of subfigures. The smoother nature of these heatmaps versus the left column is a natural consequence of being in a plane an equal distance from both planes of iron atoms.

**Figure 3:**
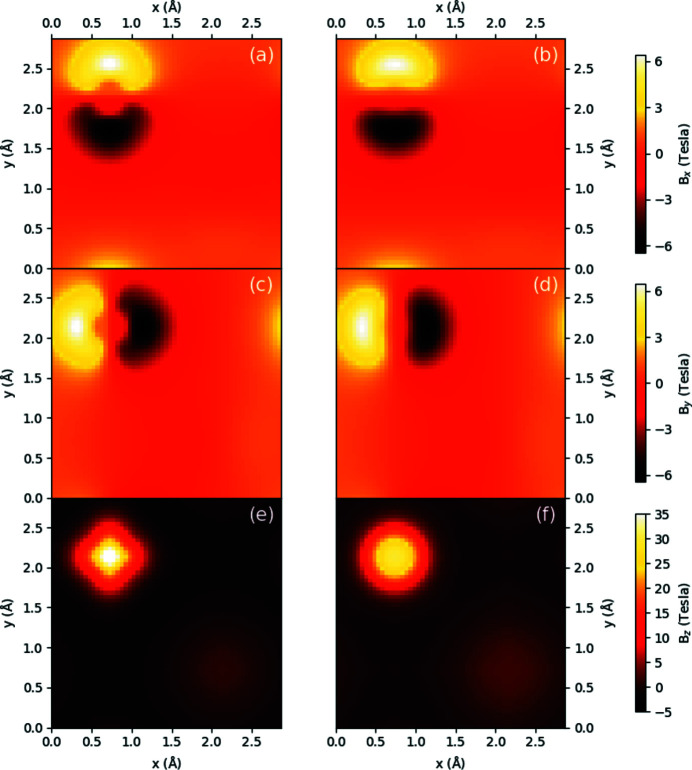
Density plots of a b.c.c. iron unit cell for (*a*), (*b*) 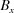 , (*c*), (*d*) 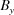 , (*e*), (*f*) 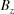 in the *xy* plane 0.25 Å above one of the iron atoms, with (*a*), (*c*), (*e*) calculated directly from the DFT-calculated spin density and (*b*), (*d*), (*f*) calculated via the parameterized values shown in Table 1 ▸ for the iron atom, with magnetic moments normalized to bulk values.

Aside from the performance of the parameterized magnetism approach in generating magnetic vector potentials and fields over large supercells, it is instructive to see the predictive capabilities of this approach by contrasting with **A** and **B** generated directly from the DFT supercell. Figs. 3 ▸ and 4 ▸ show density plots of the three components of **B** in the *xy* plane for a periodic b.c.c. iron unit cell and a periodic FePt unit cell, respectively, with all moments aligned along the *z* axis. Both sets of subfigures evaluate the magnetic fields at a *z*-axis location 0.25 Å above the topmost iron atom, with the left column showing results from the converged spin density of a DFT calculation over a unit cell and the right column showing the parameterized magnetization approach, including the scaling of **A** and **B** by the bulk moments as listed in Section 2.3.

For Fig. 3 ▸, the approximations of the magnetic fields along the *x* and *y* directions are in close agreement both in magnitude and shape. Along the *z* direction, the density map in Fig. 3 ▸(*f*) reveals the underlying symmetry inherent in the quasi-dipole approximation, as a purely spherical shape surrounds the atom, in contrast to Fig. 3 ▸(*e*). In both (*e*) and (*f*) the presence of the second iron atom in the bottom right can also be faintly seen. As the magnitudes along this direction are also in close agreement, it is likely that the parameterized magnetization approach will serve as a close approximation to the magnetic behaviour for this system.

**Figure 4:**
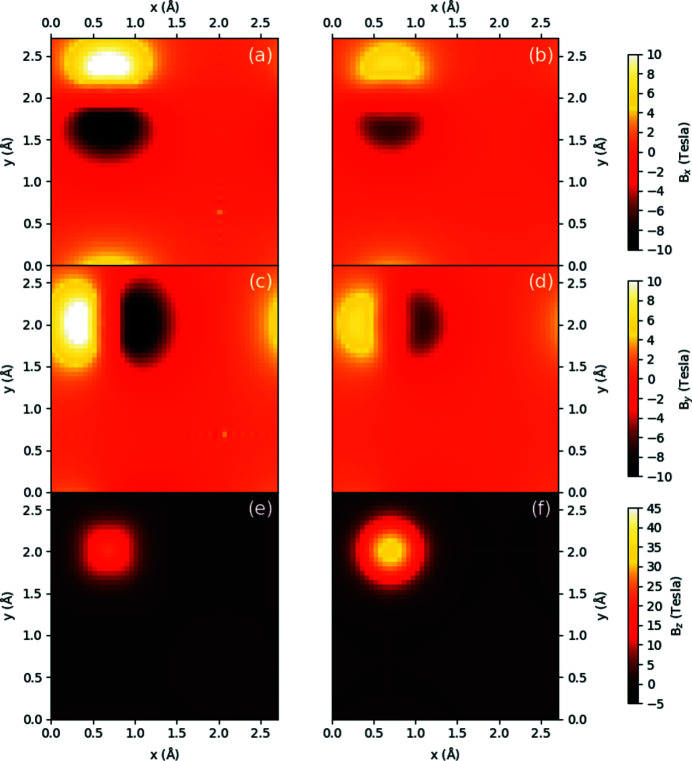
Density plots of a FePt unit cell for (*a*), (*b*) 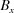 , (*c*), (*d*) 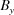 , (*e*), (*f*) 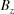 in the *xy* plane 0.25 Å above the iron atom, with (*a*), (*c*), (*e*) calculated directly from the DFT-calculated spin density and (*b*), (*d*), (*f*) calculated via the parameterized values shown in Table 1 ▸ for the iron and platinum atoms, with magnetic moments normalized to bulk values.

For Fig. 4 ▸, we again see qualitatively that the general shape along all directions for the **B** field are in good agreement, with the rightmost column showing parameterized magnetic fields having a more symmetrical character than their DFT counterparts. However, in contrast to b.c.c. iron in Fig. 3 ▸, it is noticeable that along the *x* and *y* directions the PM approach underestimates the DFT field by a factor of 0.6, while along the *z* direction the **B** field is overestimated by a factor of 1.55. For this material, deformation of the electron density surrounding the iron atoms in response to neighbouring platinum atoms has changed the surrounding magnetic vector potentials and fields in such a way that our quasi-dipole approximation, which enforces a fixed ratio between the three directions of each quantity, cannot provide a suitable quantitative fit along every direction. It is expected that if calculations within the multislice method are especially sensitive to these ratios, the results for the parameterized magnetization approach may provide a quantitatively worse description for the magnetic properties of the system. The consequences of this anisotropy of spin density will be evaluated in Section 3.3 below.

**Figure 5:**
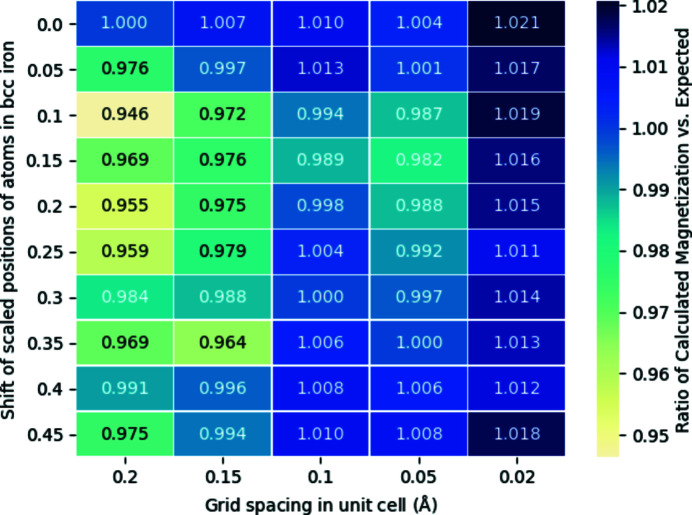
Heatmap of the ratio of the calculated total magnetization versus the expected for a collinear unit cell of b.c.c. iron, using grid spacings ranging from 0.02 to 0.2 Å and with the locations of iron atoms located in the scaled coordinates ranging between 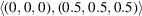 (shift of 0) and 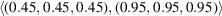 (shift of 0.45).

Returning to a point made in Section 2.2, we explore numerical behaviour in utilizing the parameterized magnetism approach with regards to grid sizes and sparsity. For the first aspect, as the parameterization for each atom is calculated and fitted on a 0.11 Å-spaced grid, it is expected that grids of different sizes, especially coarser ones, may have a substantial effect on the total magnetization within a cell. The second aspect regarding sparsity relates to the mismatch between unit-cell parameters and the grid spacing, as for sparser grids the fields around each atom will not be sampled as evenly or as symmetrically as for a fine grid. In Fig. 5 ▸, calculations of **A** and **B** over 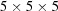 supercells of collinear b.c.c. iron were done with the parameterized magnetism approach, with the unit cells varying between those with atoms located at 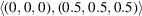 in scaled coordinates and those at 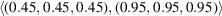 in steps of 0.05. Grids of size 0.02, 0.05, 0.1, 0.15 and 0.2 Å were used for each system. The expected magnetization per unit cell is given by the two iron atoms each with local magnetic moment of 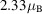 .

Two main features stand out in Fig. 5 ▸. First, for a grid spacing lower than 0.1 Å, the total magnetization per unit cell of b.c.c. iron will be within 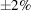 of the expected value, reflecting the smoothness of the quasi-dipole approximation and its portability to fine grids of different sizes. It is worth noting that the higher magnetization ratio for the finer grid is not necessarily the case across the parameterization of every element and should be considered on a case-by-case basis. Second, as expected, sparse grids have a strong influence both on the magnetization ratio relative to the finer grids and between grids of the same size but with geometrically isomorphic unit cells. Most importantly, the choice of a suitably fine grid for calculations of the magnetic vector potential and fields should yield a magnetization in line with calculations optimized on the atomic DFT grid.

### 3.3. Magnetic signal in the multislice method using parameterized magnetism   

Most important for the parameterized magnetism approach, from the perspective of performing multislice calculations, is its predictive capability for the magnetic signal for large supercells. For the case where the magnetic moments in a large supercell all point in different directions, doing DFT simulations to determine the magnetic field across the entire cell is challenging computationally. However, for a fully collinear system, the periodic magnetic vector potential and field in any one unit cell will be identical, while the non-periodic component can be computed via equation (7), and the results of multislice calculations from both DFT and parameterized magnetism can be directly compared.

**Figure 6:**
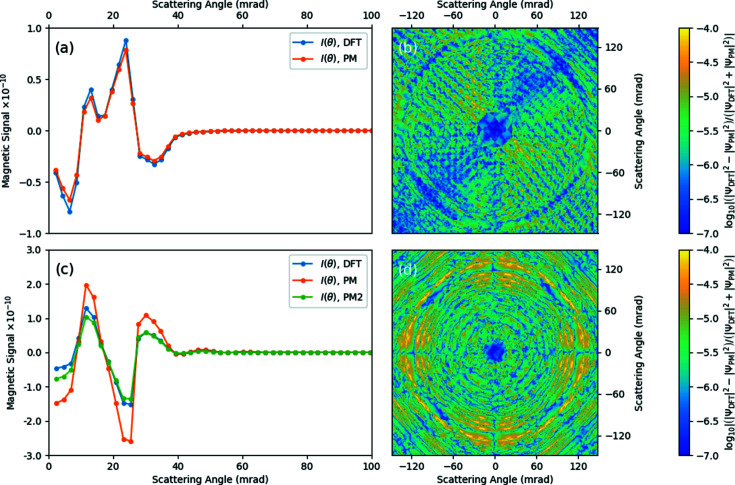
Magnetic signal for collinear (*a*) b.c.c. iron and (*c*) FePt using magnetic vector potentials and fields calculated using DFT and PM, respectively, with PM2 for the FePt showing the magnetic signal with PM parameters scaled directly using DFT fields instead of bulk magnetic moments. The logarithms of the relative ratio of the squared amplitude of the output wavefunctions for the DFT and PM methods are given for (*b*) b.c.c. iron and (*d*) FePt. Output wavefunctions are calculated with the Pauli multislice method (Edström *et al.*, 2016*b* ▸) using 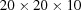 unit supercells with 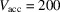 kV and α = 25 mrad.

For Fig. 6 ▸, supercells consisting of 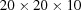 unit cells for both b.c.c. iron (a 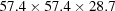 Å supercell) and FePt (a 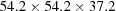 Å supercell) were considered. An acceleration voltage of 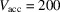 kV was used along with a convergence semi-angle of α = 25 mrad. Debye–Waller factors have not been included in order to allow for a one-to-one comparison of the parameterization method with DFT calculations, although they can generally be applied within the Pauli multislice scheme. A magnetic field of 2 T was added to both crystals. Multislice calculations were performed with zero periodic magnetic components (*i.e.* all moments set to zero) for calibration, with the DFT-calculated magnetic fields for b.c.c. iron and FePt as outlined in Section 2.3, and with PM calculations done using the values of bulk moments obtained from DFT as listed in Section 2.3. In addition, for the FePt, calculations were also carried out with the tabulated values for iron normalized instead by 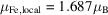 and 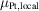 = 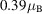 . This is done in order to explore the effect of having the 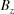 for the FePt system with moments aligned along the *z* axis match quantitatively with the DFT results as seen in Fig. 3 ▸. This approach has been labelled as PM2 in Fig. 6 ▸.

Plotted in subfigures (*a*) and (*c*) of Fig. 6 ▸ are the radial magnetic signal after subtracting the squared amplitude of the calibration exit wavefunction, 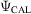 , at each pixel from that of the calculated exit wavefunction, given by 

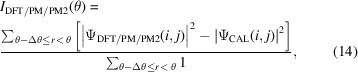

where 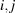 are the pixel positions relative to the centre of the diffraction pattern, 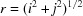 , ‘CAL’ refers to the calibrated exit wavefunction from only having the 2 T field applied to the supercell, and a 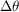 of 2 mrad was chosen. Both subfigures reveal that the PM approach is to a strong degree able to qualitatively predict the magnetic signal in these large supercells to a similar degree as DFT-generated magnetic vector potentials and fields across all areas of the diffraction pattern. For the b.c.c. iron in (*a*), the PM consistently underestimates the DFT magnetic signal by a factor between 5 and 10%, matching the expectation hinted at in Fig. 3 ▸ that the two magnetic quantities were qualitatively and quantitatively similar. For FePt in (*c*), the PM consistently overestimates the magnetic signal relative to DFT, with the smallest scattering angles especially showing a difference of the order of 200% difference. For this reason the PM2 approach is included, recalibrating the magnetic moments so that the 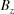 fields are in close quantitative agreement. The PM2 approach clearly works to bring the magnetic signal more in line with the DFT-predicted value, suggesting that this may be a superior approach for systems with strong deformation of the electronic density around the atoms concerned. (*b*) and (*d*) in Fig. 6 ▸ show the logarithm of the relative ratio (*i.e.* difference divided by the sum) of the squared amplitude of the DFT and PM exit wavefunctions for the b.c.c. iron and FePt supercells, respectively, providing a visual clue as to the degree to which these two approaches are in agreement, with the b.c.c. iron relative ratio consistently below 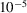 across the whole diffraction pattern while the FePt relative ratio does not go beyond 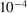 . While the relative ratio appears highest beyond 60 mrad from the centre, it should be noted that the sample thickness in these simulations is approximately 3 nm, meaning that most of the intensity in the diffraction pattern is concentrated in the central disc and that scattering intensities outside the central disc are much smaller and therefore more sensitive to minute changes in the intensity.

## 4. Conclusion   

A framework for the atomic parameterization of magnetic vector potentials and fields for transition metal elements has been presented herein, with the overarching goal being to provide an efficient and reliable method for the inclusion of magnetic effects in magnetic multislice calculations (Edström *et al.*, 2016*b* ▸) for materials and crystals of arbitrary size. Calculating these magnetic quantities traditionally requires either a heavy effort on the part of computationally demanding software, or on locally inaccurate approximations like a classical dipole method (Jackson, 1999 ▸). Relying on spin densities generated in *GPAW* (Mortensen *et al.*, 2005 ▸; Enkovaara *et al.*, 2010 ▸), a quasi-dipole approximation consisting of ten free parameters was fitted using least-squares for 25 transition metal elements (Newville *et al.*, 2021 ▸). The flexibility of this approach was showcased by a magnetic vector potential **A** on a grid of size 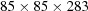 Å for a b.c.c. iron supercell with moments aligned according to spatially correlated normal and uniform distributions. The performance of the parameterized magnetization was directly compared with magnetic quantities derived from DFT calculations in the unit cell for b.c.c. iron and for tetragonal FePt, showing that the performance of the parameterization is best for materials without significant deformation of their spin density due to bonding (Billas *et al.*, 1994 ▸). The performance of the parameterized magnetization approach was shown to be flexible on grids and geometries of different sizes. Lastly, a direct comparison of the magnetic signals resulting from Pauli multislice calculations of the approach with DFT calculations showed that for both b.c.c. iron and tetragonal FePt, the parameterized magnetism method was able to capture the behaviour of the magnetic signal as a function of scattering angle, with better quantitative results depending on the scaling of magnetic moments in the unit cell.

## Figures and Tables

**Table 1:** Parameterized magnetic factors for transition metal elements from scandium (Z = 21) to gold (Z = 79), following equations (11) and (12) Elements with filled *d* orbitals or for which *GPAW* has no atomic PAW setups are left out. The parameters a_{i} are scaled so that the resulting **A** and **B** values correspond to a spin magnetic moment of one Bohr magneton (μ_B_). The RMS error is included for each calculation.

	Pair 1		Pair 2		Pair 3		Pair 4		Pair 5		RMS error	
Element	a_{0} (T Å^3^)	b_{0} (Å^3^)	a_{1} (T Å^3.5^)	b_{1} (Å^3.5^)	a_{2} (T Å^4^)	b_{2} (Å^4^)	a_{3} (T Å^4.5^)	b_{3} (Å^4.5^)	a_{4} (T Å^5^)	b_{4} (Å^5^)	**A** (T Å)	**B** (T)
Sc	9.627E-01	1.298E+00	−1.195E-02	1.887E-03	3.207E-03	7.223E-04	1.746E-01	2.624E-01	2.062E-02	3.740E-02	7.075E-03	2.531E-03
Ti	9.865E-01	2.000E+00	−2.565E-03	1.442E-03	3.906E-01	2.528E-01	−5.238E-01	6.228E+00	−1.731E-01	3.869E-01	1.416E-02	4.423E-02
V	6.594E+00	4.638E+02	2.007E+00	4.761E-01	−4.102E+01	3.962E+03	−1.165E+00	2.720E+00	−7.793E-01	4.911E-01	1.807E-02	1.154E-02
Cr	1.201E+00	4.655E+00	1.304E+00	2.783E-01	−5.867E+00	1.045E+00	3.577E+00	5.462E+00	3.880E+00	1.312E+00	1.982E-02	1.625E-02
Mn	8.182E-01	4.156E+00	2.390E+00	2.663E-01	−6.858E+00	3.055E+00	6.222E+00	4.960E+00	−7.540E-01	2.450E-01	1.580E-02	2.844E-02
Fe	6.967E+00	4.357E+02	1.799E+00	1.894E-01	−4.485E+01	3.955E+03	−6.665E-01	1.294E+00	−5.191E-01	1.693E-01	1.807E-02	2.034E-02
Co	5.324E+00	2.716E+02	1.894E+00	1.638E-01	−3.158E+01	2.386E+03	−8.253E-01	9.567E-01	−5.211E-01	1.285E-01	1.781E-02	9.368E-02
Ni	2.123E+00	2.109E-01	5.423E+00	6.535E+00	−4.232E+01	9.541E-01	7.010E+01	1.139E+00	−3.031E+01	1.325E+00	1.126E-02	1.231E-02
Cu	1.684E+00	1.976E-02	−1.093E+00	5.604E-02	−7.235E-02	2.058E-03	−1.153E-01	4.499E-03	−4.434E-01	5.774E-01	5.049E-03	4.416E-03
Y	1.808E+00	1.824E-03	−5.816E-01	9.904E-02	−1.824E+00	3.088E-04	1.045E+00	1.321E-04	−1.714E-01	5.709E-05	9.262E-03	3.086E-02
Zr	2.954E-03	3.582E-04	1.251E+00	2.098E+01	9.615E-01	5.496E+00	−1.008E-01	2.481E-01	1.946E-01	5.740E-01	1.096E-02	3.841E-02
Nb	9.850E-01	6.480E-02	3.354E-01	3.146E+00	−3.161E-01	2.979E-02	−1.071E+00	2.602E-01	6.619E-01	2.939E-01	1.509E-02	1.356E-02
Mo	1.150E+00	7.032E-01	2.098E-01	1.704E-02	−1.331E+00	3.853E-02	7.077E-01	3.072E-02	1.815E-01	1.724E-01	1.647E-02	2.066E-02
Ru	1.141E+00	7.594E-01	2.508E-01	1.774E-02	−1.329E+00	4.251E-02	5.850E-01	1.624E-01	3.849E-01	2.449E-02	1.386E-02	4.651E-02
Rh	2.283E-02	1.625E-03	2.769E+00	1.724E+00	−2.187E+00	2.573E+00	−1.444E-01	4.847E-02	1.402E-01	6.810E-02	1.379E-02	7.110E-02
Pd	7.623E-03	4.048E-04	−3.558E+00	9.647E+01	9.942E+00	1.691E+02	2.203E+00	1.102E+01	2.391E-01	4.986E-01	6.902E-03	3.193E-02
Ag	4.349E+00	3.961E+01	−8.312E+00	1.537E+02	4.611E-01	2.675E-01	−6.189E-01	3.481E-01	2.320E+00	2.111E+01	8.045E-03	4.964E-03
Hf	8.602E-01	1.201E+00	2.697E-01	6.587E-02	−1.946E-01	1.345E+00	−1.715E-01	5.218E-02	−1.149E-01	3.384E-01	1.061E-02	3.482E-02
Ta	9.782E-01	1.502E+00	2.160E-01	5.528E-02	−6.757E-01	1.135E-01	3.900E-01	1.210E+00	2.566E-01	1.225E-01	1.199E-02	3.337E-02
W	8.705E-01	1.171E+00	9.543E-01	6.933E-02	−1.546E+00	7.871E-02	2.710E-01	2.382E-01	3.197E-01	5.784E-02	2.105E-02	1.434E-02
Re	2.070E+00	6.697E+02	1.518E+00	9.766E-02	4.992E-01	1.397E+01	−5.630E-01	4.738E-02	−4.218E-01	2.385E-01	1.641E-02	2.250E-02
Os	1.042E+00	1.469E+00	3.499E-01	5.149E-02	−1.514E+00	1.153E-01	1.086E+00	9.417E-01	7.026E-01	1.142E-01	1.373E-02	3.353E-02
Ir	2.514E-02	2.202E-02	1.139E+01	1.011E+02	−2.154E+01	2.992E+02	2.050E+00	6.562E+00	−2.924E-02	4.927E-02	1.846E-02	1.204E-02
Pt	2.001E+00	1.925E-01	−3.305E+00	2.058E-01	1.185E+00	8.158E+00	3.716E+00	2.857E-01	−2.088E+00	3.153E-01	6.134E-03	4.514E-02
Au	6.100E-01	1.747E+00	1.069E+00	2.511E+00	6.258E-01	2.764E-01	−3.433E+00	6.340E-01	2.102E+00	7.760E-01	7.167E-03	5.947E-03
